# Circulating microRNA-636 is associated with the elimination of hepatitis C virus by ombitasvir/paritaprevir/ritonavir

**DOI:** 10.18632/oncotarget.25889

**Published:** 2018-08-10

**Authors:** Asahiro Morishita, Hirohito Yoneyama, Hisakazu Iwama, Koji Fujita, Miwako Watanabe, Kayo Hirose, Tomoko Tadokoro, Kyoko Oura, Teppei Sakamoto, Shima Mimura, Takako Nomura, Makoto Oryu, Takashi Himoto, Kunitada Shimotohno, Tsutomu Masaki

**Affiliations:** ^1^ Department of Gastroenterology and Neurology, Ikenobe Miki-cho, Kita-gun, Kagawa 761-0793, Japan; ^2^ Life Science Research Center, Ikenobe Miki-cho, Kita-gun, Kagawa 761-0793, Japan; ^3^ Department of Internal Medicine, Kagawa Saiseikai Hospital, Tahikamimachi, Takamatsu, Kagawa 761-8076, Japan; ^4^ Department of Medical Technology, Kagawa Prefectural University of Health Sciences, Hara, Mure-cho, Takamatsu, Kagawa 761-0123, Japan; ^5^ Research Center for Hepatitis and Immunology, National Center for Global Health and Medicine, Kohnodai, Ichikawa, Chiba 272-8516, Japan

**Keywords:** circulating microRNA, microRNA-636, ombitasvir/paritaprevir/R, HCV elimination, direct-acting antiviral

## Abstract

Hepatitis C virus (HCV) infection causes sustained inflammation and fibrosis. Several oral direct-acting antivirals (DAAs) including ombitasvir/paritaprevir/ritonavir (OBV/PTV/r) were recently developed for HCV elimination. The combination of DAAs brought a higher sustained viral response (SVR) rate to anti-HCV therapy compared to interferon (IFN)-based regimens. However, 5% of hepatitis C patients who undergo DAA therapy still suffer from a sustained HCV infection. MicroRNA (miRNA) is essentially interfering, endogenous noncoding RNA that has been investigated as a new biomarker for the response to DAA in hepatitis C patients. Here we used a miRNA array and real-time polymerase chain reaction (PCR) to determine the targetable miRNA before and 12 weeks after OBV/PTV/r treatment for refractory hepatitis C. We used replicon cells, in which genotype 1b type HCV is stably transfected in Huh7 cells, to determine whether miRNA can inhibit HCV replication. Among 2,555 miRNAs, three were significantly up-regulated and eight miRNAs were down-regulated in serum 12 weeks after OBV/PTV/r treatment. An unsupervised hierarchical clustering analysis, using Pearson's correlation, showed that the miRNA profiles between before and 12 weeks after OBV/PTV/r treatment were clustered separately. At 12 weeks after OBV/PTV, miR-636 was targeted among the eight down-regulated miRNAs, and the expression level of circulating miR-636 was significantly diminished. The amount of HCV-RNA was significantly diminished 48 hours after miR-636 inhibitor transfection in HCV replicon cells. In conclusion, miR-636 might be one of the essential targetable molecules in HCV patients who undergo DAA therapy and still suffer from a sustained HCV infection.

## INTRODUCTION

Hepatitis C virus (HCV) is one of the most damaging viruses [1, 2], and its chronic infection causes sustained inflammation and fibrosis [3, 4]. Until recently, interferon (IFN)-based regimens had been used to treat HCV infection, and anti-HCV therapy was restricted due to its severe side effects, resulting in a poor cure rate [5, 6]. Several oral direct-acting antivirals (DAAs) for HCV elimination were developed over the last several years [7–10]. The combination of DAAs brought a revolutionary improvement to anti-HCV therapy. Twelve-week DAA combination therapy eliminates HCV with very high probability and without noticeable side effects. The sustained viral response (SVR) rate in chronic HCV genotype 1b (HCV GT-1b) infection is now reported to be 95%–100% [10]. However, approx. 5% of HCV patients who undergo DAA therapy still suffer from a sustained HCV infection.

Ombitasvir, dosed once daily, is an HCV NS5A inhibitor that acts by inhibiting the HCV protein NS5A. Paritaprevir, an HCV NS3/4A protease inhibitor that decreases the expression of HCV NS3/4A protein, is administered with low-dose ritonavir (paritaprevir/ritonavir) to enhance the patient's paritaprevir plasma levels and prolong its half-life. Both ombitasvir and paritaprevir have powerful *in vitro* antiviral activity for multiple subtypes of HCV, including 1a, 1b, 2a, 2b, 3a, 4a, and 6a. A randomized phase III trial of OBV/PTV/r revealed that the SVR rate at 12 weeks post-treatment was 94.9% in HCV genotype 1b-infected patients [8]. However, virological failure occurred in 3.0% of the HCV patients treated with OBV/PTV/r in that trial [8]. In order to reduce the virological failure rate, it is important to determine the precise mechanisms that underlie the elimination of HCV by OBV/PTV/r directly and indirectly, including the regulation of miRNA in host cells.

MicroRNAs (miRNAs) are essentially 18–22-nucleotide-long, interfering, endogenous noncoding RNAs, and more than one thousand of miRNAs have been discovered in the human genome [11]. The effect of miRNAs on the regulation of the expression of various genes is so broad that one miRNA promotes the targeting and modulation of >200 genes [12]. The HCV genome is a positive-sense, single-stranded RNA with a conserved 5' noncoding region (NCR), one open reading frame (ORF) and a conserved 3'-NCR. The single ORF encodes a polyprotein, and its N terminus is cleaved by endoplasmic reticulum (ER) signal peptidase and/or signal peptide peptidase (SPP) into three structural proteins (core, E1, and E2) [13]. The other sequence of the polyprotein is processed by the viral NS2 and/or NS3/4A protease into seven nonstructural proteins (p7, NS2, NS3, NS4A, NS4B, NS5A and NS5B), which play important roles in the HCV life cycle [14].

Interestingly, the HCV genome does not encode viral miRNA [15, 16]. However, HCV infection alters the expression of host miRNAs during the progression of liver disease such as liver fibrosis, cirrhosis, and hepatocellular carcinoma [17–20]. In fact, host miRNAs control the HCV life cycle by directly binding to HCV RNAs or indirectly targeting cellular mRNAs [13]. Increasing evidence indicates that miRNAs are one of the centered factors in the interaction network between a virus and a host [13]. In addition, HCV RNA segregates host miRNAs from their normal host targets and reduces host gene expression, resulting in a persistent HCV infection [21]. This interference by HCV RNA might be a key to elucidate the mechanism of HCV elimination by OBV/PTV/r.

Therefore, the modulation of miRNAs, which are associated with the elimination process by OBV/PTV/r, might be valuable therapeutic targets in anti-HCV therapy. In this study, we identified the targetable miRNAs which are related to the HCV elimination process brought about by OBV/PTV/r.

## RESULTS

### Sample characteristics

As shown in Table 1, 11 males and seven females were characterized. Of these, nine patients were ≤70 and nine patients were >70 years old (mean: 70.5±8.3 yrs). Thirteen of the HCV-positive patients had chronic hepatitis, and five of them had liver cirrhosis as a histological background. Among the 18 patients with chronic hepatitis C, the ALT levels were ≤40 IU/L in 14 patient and >40 IU/L in four patients (mean: 70.5±8.3 IU/L). The estimated glomerular filtration rate (eGFR) was <30 mL/min/1.73 m^2^ in six patients, 30–50 mL/min/1.73 m^2^ in four patients, and >50mL/min/1.73 m^2^ in eight patients (mean: 37.1±26.1). Nine patients showed <6 HCV RNA, and nine patients showed ≥6 HCV RNA (log IU/mL, mean 6.0±0.5).

**Table 1:** Clinicopathological features of patients with chronic hepatitis C (n=18, genotype Ib, Y93H wild)

Age (year, mean 70.5±8.3):	
≤70	9
>70	9
Sex:	
F	7
M	11
Histological background:	
CH	13
LC	5
ALT (IU/L, mean 70.5±8.3):	
≤40	14
>40	4
eGFR (mL/min/1.73 m2, mean 37.1±26.1):	
<30	6
30–50	4
>50	8
HCV-RNA (Log IU/mL, mean 6.0±0.5):	
<6	9
≥6	9

### Response to OBV/PTV/r treatment

**Figure 1:**
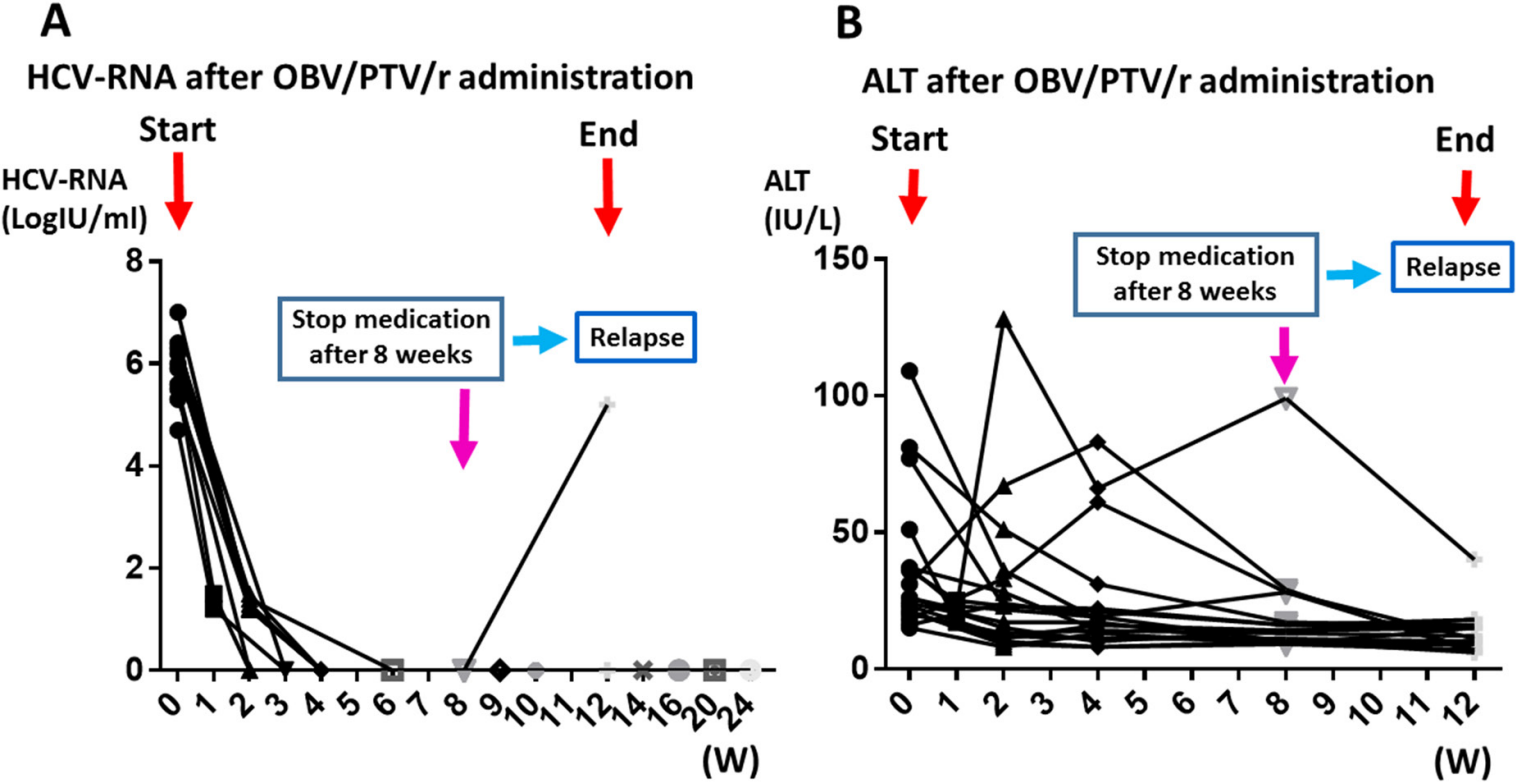
Clinical features during OBV/PTV/r treatment **(A)** Changes in the amount of HCV-RNA during OBV/PTV/r treatment. **(B)** Changes in the level of ALT during OBV/PTV/r treatment. One patient experienced a grade 4 side effect at week 8 and failed to complete the OBV/PTV/r treatment.

Of the 18 patients who received OBV/PTV/r treatment, 17 had serum samples collected for miRNA analysis at time points before and at week 4, week 6, and week 12 (One serum sample, which is from relapse case was excluded). All patients were infected with HCV genotype 1 with no genetic mutation in NS5A lesion. The sustained virological response rate at 4 weeks after the start of treatment (SVR_4_) was 94.4%, and that at 6 weeks (SVR_6_) of treatment was 100% (Figure 1A). The SVR_12_ was 94.4% in the treatment-naïve patients who received OBV/PTV/r treatment (Figure 1A). In addition, the normalization rates of ALT after 4 weeks and 6 weeks were 83.3% and 88.9%, respectively (Figure 1B). One patient experienced a grade 4 side effect at week 8 and failed to complete the OBV/PTV/r treatment (Figure 1A).

### Differences in miRNA in the serum samples obtained between before and after OBV/PTV/r treatment

**Figure 2:**
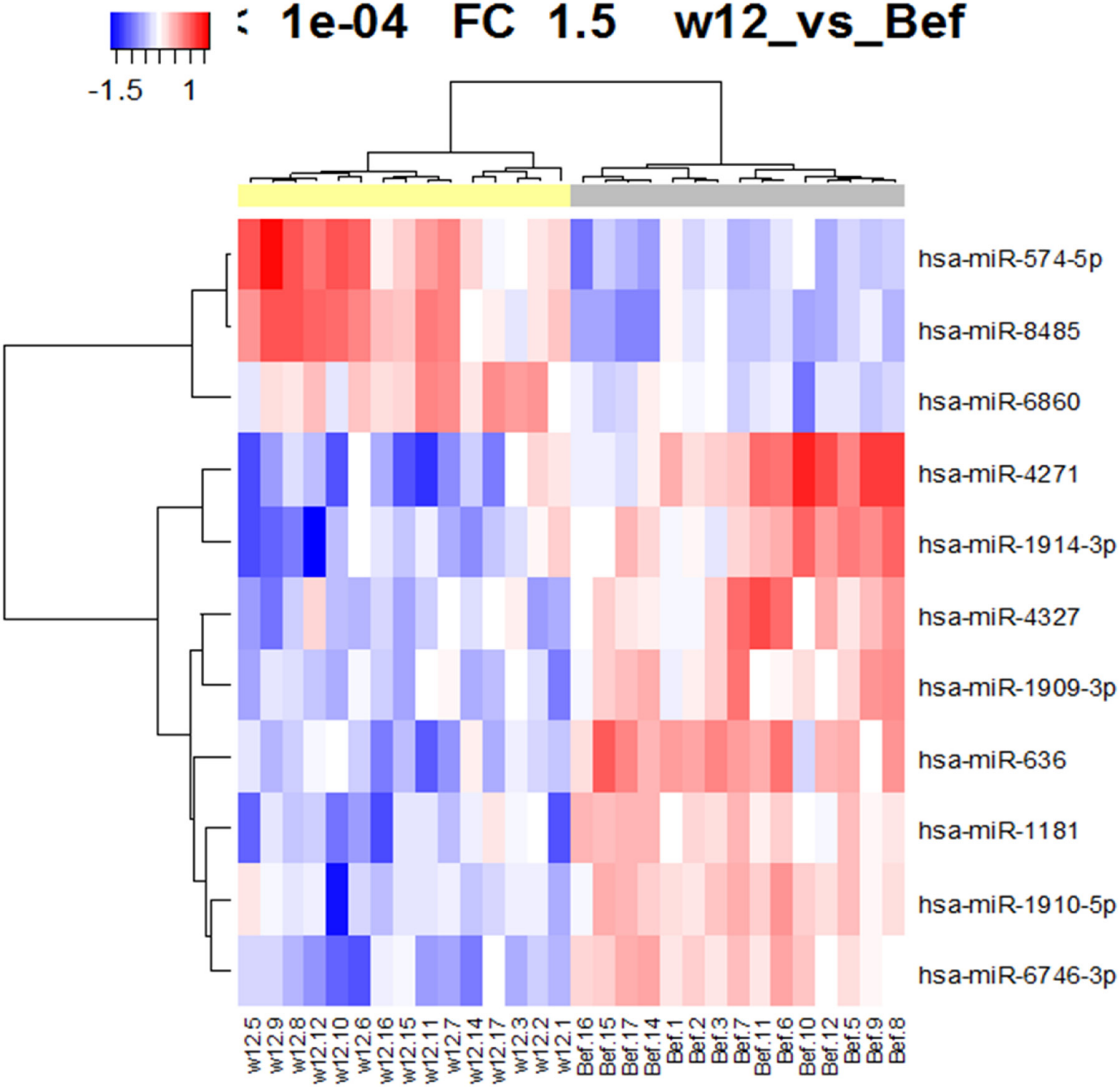
Hierarchical clustering of the 11 miRNAs expressed between before and 12 weeks after the start of OBV/PTV/r treatment (p<0. 0001)

Using a custom microarray platform, we analyzed the expression levels of 2,555 human miRNA probes in the patients' serum samples obtained before and after the start of OBV/PTV/r treatment. As shown in Figure 2, Table 2, and Table 3, our comparison of the expression of miRNAs in serum samples between before and after the start of OBV/PTV/r treatment revealed that at 12 weeks after the start of OBV/PTV/r treatment, of the 2,555 miRNAs just three miRNAs were significantly up-regulated in the sera (Figure 2, Table 2) and eight were down-regulated (Figure 2, Table 3). The unsupervised hierarchical clustering analysis using Pearson's correlation showed that miRNA profiles between before and at 12 weeks after the start of OBV/PTV/r treatment were clustered separately (Figure 2).

**Table 2:** Three microRNAs were significantly up-regulated 12 weeks after OBV/PTV/r administration (p<0.0001)

	P-value	FC (W12/Bef)	FDR
**hsa-miR-574-5p**	9.0E-08	2.10	2.7E-05
**hsa-miR-8485**	8.2E-06	2.05	7.0E-04
**hsa-miR-6860**	6.3E-05	1.51	1.9E-03

**Table 3:** Eight microRNAs were significantly down-regulated 12 weeks after OBV/PTV/r administration (p<0.0001)

	p-value	FC (W12/Bef)	FDR
**hsa-miR-6746-3p**	5.2E-08	0.58	2.7E-05
**hsa-miR-1181**	1.3E-06	0.63	1.9E-04
**hsa-miR-1910-5p**	5.6E-06	0.65	6.0E-04
**hsa-miR-1914-3p**	1.2E-05	0.54	8.0E-04
**hsa-miR-636**	1.2E-05	0.51	8.0E-04
**hsa-miR-1909-3p**	3.0E-05	0.64	1.6E-03
**hsa-miR-4271**	6.2E-05	0.45	1.9E-03
**hsa-miR-4327**	8.1E-05	0.59	2.1E-03

### Circulating miR-636 expression in serum is down-regulated 12 weeks after the start of OBV/PTV/r treatment

**Figure 3:**
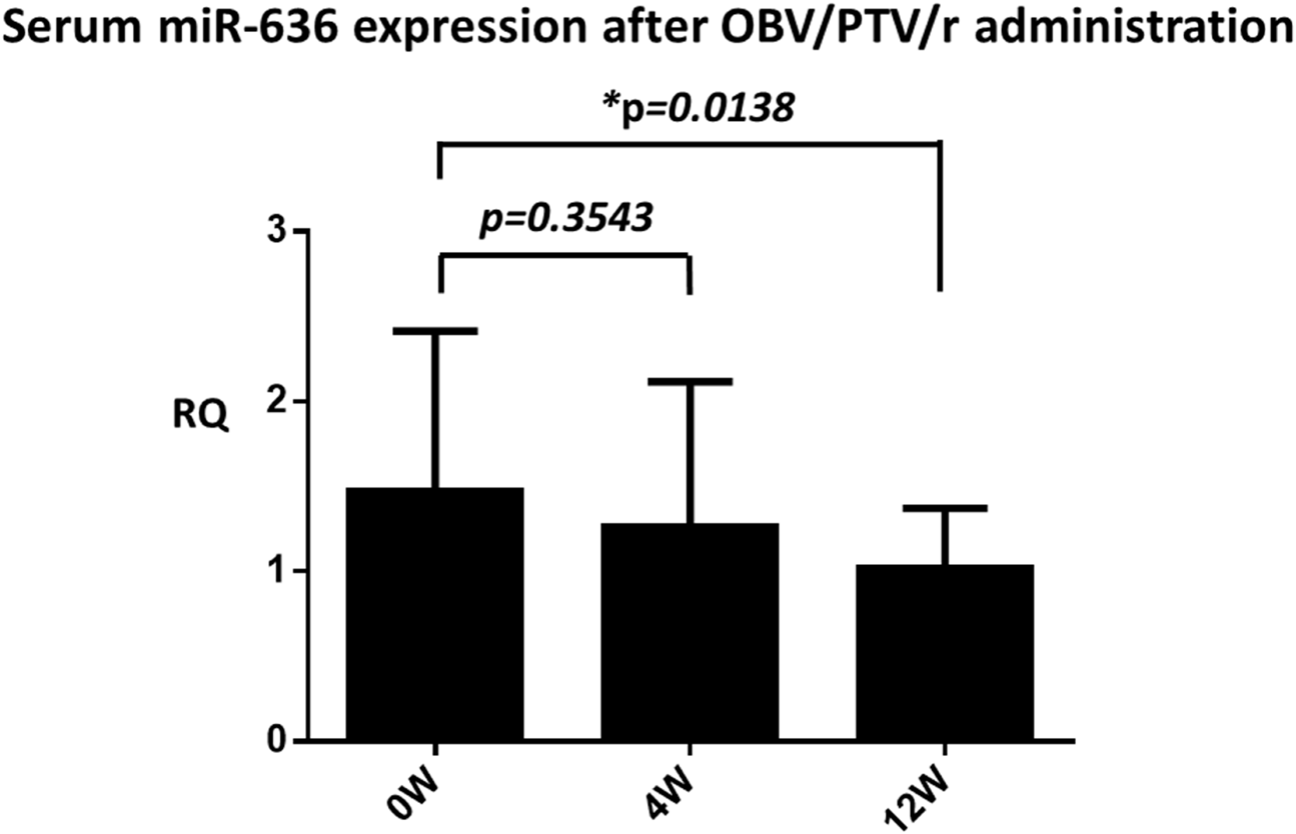
Serum miR-636 expression before, 4 weeks, and 12 weeks after the start of OBV/PTV/r treatment miR-636 was significantly diminished after 12 weeks compared to before OBV/PTV/r treatment. ^*^p<0.05.

To elucidate the target miRNA related to the elimination of HCV by OBV/PTV/r, we targeted miR-636 among the eight down-regulated miRNAs. We determined the expression level of circulating miR-636 using serum samples and real time RT-PCR at time points before and at week 4 and week 12 after the start of OBV/PTV/r treatment. Remarkably, miR-636 was significantly diminished 12 weeks after the start of OBV/PTV/r treatment (Figure 3).

### Inhibition of miR-636 reduces HCV RNA replication

**Figure 4:**
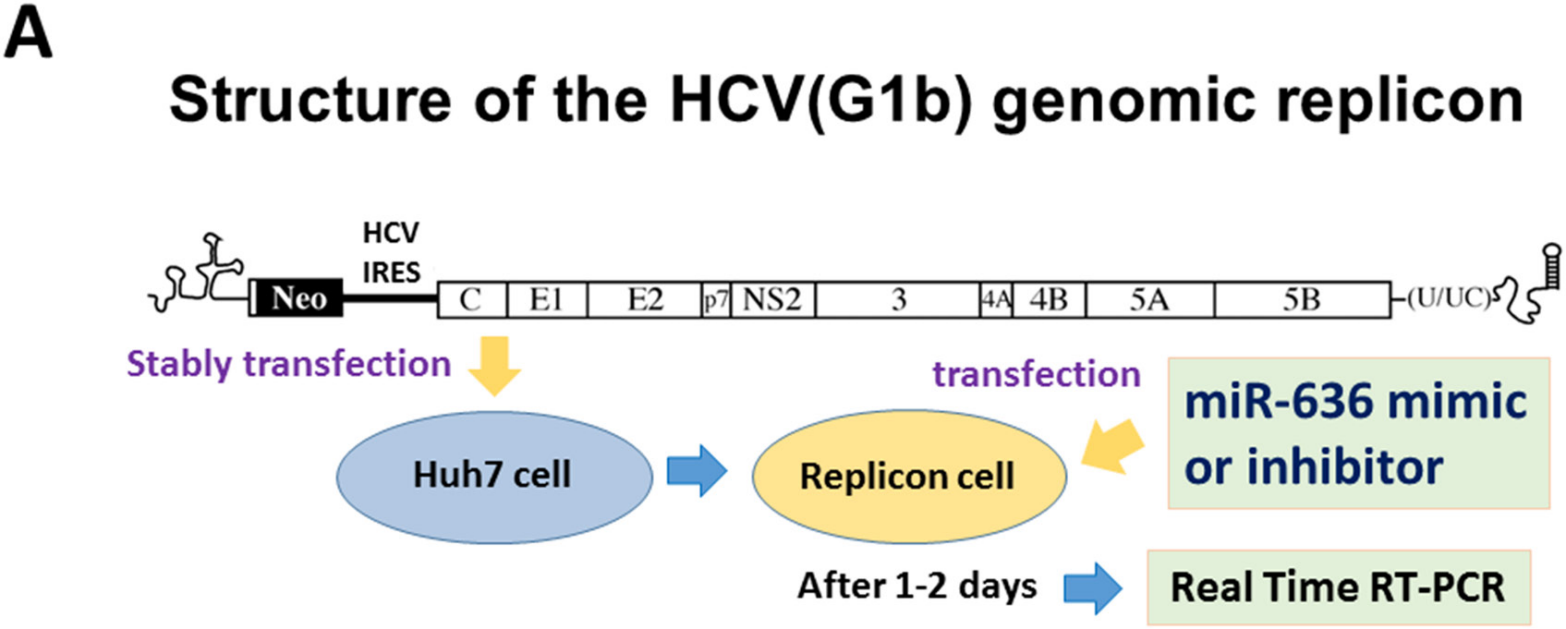

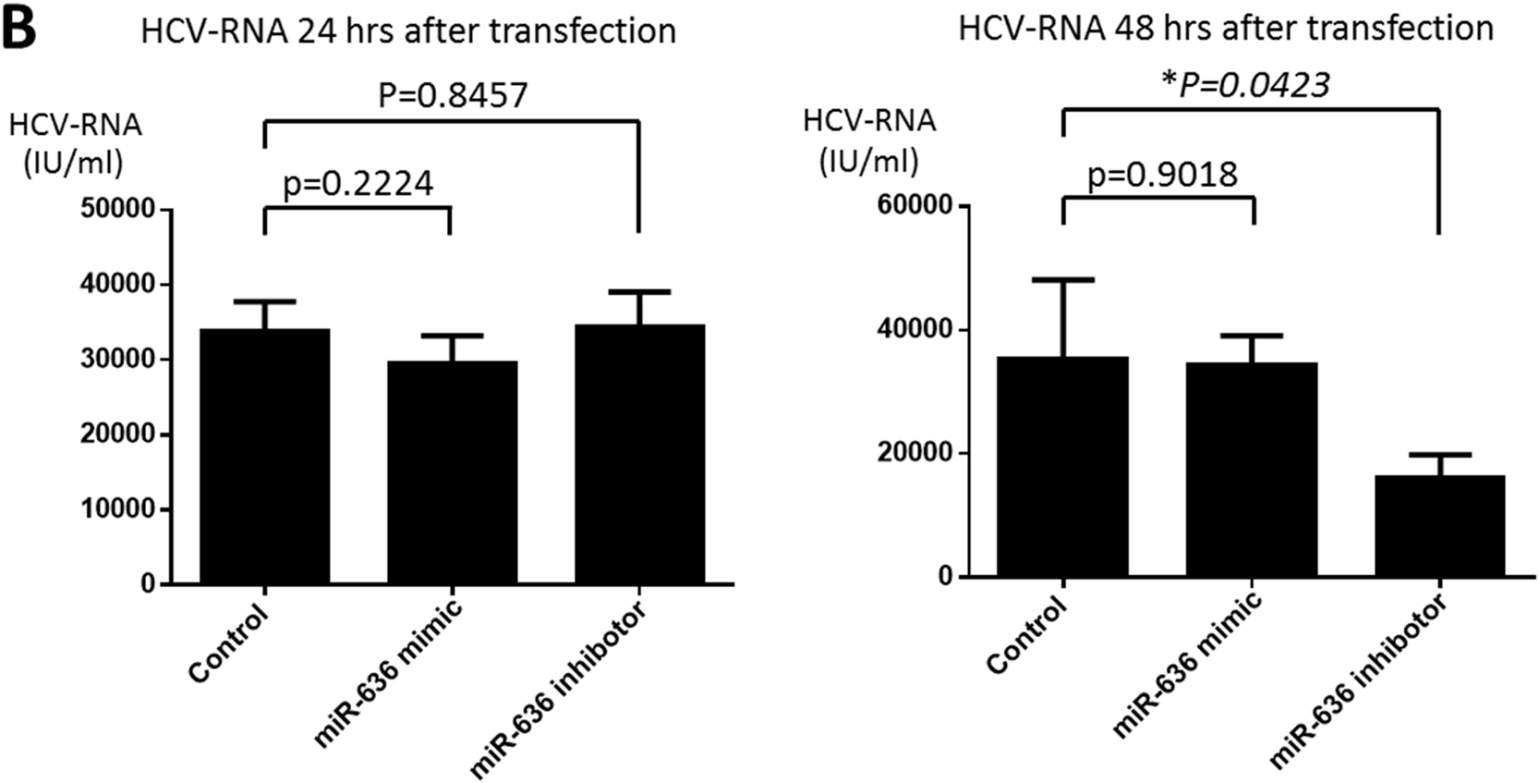
Loss of miR-636 expression reduced the HCV-RNA replication in Huh7 cells with HCV GT-1b genomic replicon **(A)** Schematic of the structure of the HCV GT-1b genomic replicon. **(B)** The amount of HCV-RNA at 24 and 48 hours after miR-636 mimic and inhibitor transfection.

Our *in vivo* study led us to hypothesize that OBV/PTV/r might inhibit HCV replication via miR-636 down-regulation. To examine this hypothesis, we used replicon cells, with which genotype 1b type HCV is stably transfected in Huh7 cells [22]. Replicon cells were transfected with miR-636 mimic, miR-636 inhibitor, or negative control miRNA. After 24 hour and 48 hour incubations, we analyzed the miR-636 and HCV RNA. Interestingly, the amount of HCV RNA was significantly diminished 48 hour after miR-636 inhibitor transfection (Figure 4B), which is in consistent with the inhibition of miR-636 (Figure 4B), although no remarkable reduction of HCV RNA was detected 24 hour after miR-636 inhibitor transfection (Figure 4B). On the other hand, the HCV RNA level was not influenced by miR-636 overexpression at 24 hour or 48 hour after miR-636 mimic transfection (Figure 4B).

## DISCUSSION

HCV genotyping is a major cause of DAA treatment failure [23]. DAAs inhibit various parts of HCV protein including NS3/4A, NS5A, and NS5B protein. Gene mutations of these loci lead to the treatment failure by different types of DAAs. As virological failure occurred in 3.0% of HCV patients treated with OBV/PTV/r [8], HCV NS5A and HCV NS3/4A mutations may exist in OBV/PTV/r-failure patients. It is refractory for the patients with mutant HCV to eliminate HCV by OBV/PTV/r therapy.

Liver-specific miRNA miR-122 or interference of the RNA interference pathway associated with miRNA biogenesis resulted in the inhibition of HCV replication [24]. Circulating miRNAs (cmiRNAs) in the plasma or serum have been assessed for evaluations of the therapeutic response and antiviral effects in patient with hepatitis C, since cmiRNAs are protected from RNase and stabilized even in the serum [23, 25, 26]. Therefore, toward the goal of reducing the virological failure rate of DAA treatment, we examined targetable miRNAs during HCV elimination by OBV/PTV/r. Our analyses revealed three up-regulated miRNAs and eight down-regulated miRNAs at 12 weeks after the start of OBV/PTV/r treatment. Among these miRNAs, miR-636 was significantly reduced by 12 weeks of OBV/PTV/r treatment in the serum of HCV patients. Additionally, a time-lag was observed between HCV elimination and miR-636 down-regulation (Figure 3).

These results indicate that miR-636 might be involved in not only HCV elimination but also liver regeneration. Jang et al. demonstrated that a down-regulation of miR-636 is associated with cell proliferation through an enhanced expression of Ras, which is one of the putative targets of miR-636 [27]. In addition, ANT2 suppression restores mi-636 expression, thereby downregulating RAS and inhibiting cell proliferation [27]. On the other hand, miR-636 reduction is involved in the recovery from heart failure due to dilated cardiomyopathy [28]. These reports support our finding that miR-636 is related to the process of HCV elimination and recovers liver function, normalizing ALT.

To explore the functions of miR-636, we examined whether miR-636 is involved in the inhibition of HCV replication during OBV/PTV/r treatment, and whether miR-636 is suppressed as a result of HCV elimination by OBV/PTV/r treatment. We transfected replicon cells with miR-636 mimic and miR-636 inhibitor and then analyzed the HCV-RNA expression by real-time PCR. The expression level of HCV-RNA was significantly down-regulated by miR-636 inhibition (Figure 4B). We also examined the miR-636 expression at week 4 and 6, and miR-636 expression was gradually reduced as compared to before OBV/PTV/r treatment (data not shown). These results suggest that miR-636 can suppress HCV replication and its inhibitor might be a powerful antiviral drug for patients with refractory mutant HCV. In addition, in Figure 4B, inhibition of miR-636 reduced amount of HCV-RNA at 48 hour, but not at 24 hour after miR-636 inhibitor transfection. There was a time-lag between miR-636 inhibitor transfection and reduction of HCV-RNA level. Interestingly, there is no homology between miR-636 and the 5'-UTR sequences of HCV, and thus miR-636 might regulate key molecules for HCV elimination in host cells. These indicate that miR-636 have indirect inhibitory effect on HCV replication.

It was already demonstrated that the inhibition of some miRNAs including miR-199a-5p reduces the replication of HCV via regulation of the pro-survival pathway and not a direct interaction between miRNA and HCV [29]. Some of HCV host factors, including CHUK, IKK-α, and IκB kinase, are regulated by let-7a and therefore, let-7a indirectly mediates antiviral effects [30]. In addition, miR-25 and miR-130 families repress various HCV co-factors and inhibit viral infection and replication at multiple steps [30]. These reports support our data that some miRNAs can modulate HCV replication even through indirect inhibition.

Taken together, our present findings revealed that miR-636 expression is reduced in the sera of HCV patients at 12 weeks after the start if OBV/PTV/r treatment, and the loss of miR-636 expression inhibits HCV replication in human hepatocellular carcinoma cells. Therefore, miR-636 might play an important role as a powerful therapeutic target for HCV elimination and liver regeneration.

In conclusion, miR-636 is associated with the elimination process of OBV/PTV/r treatment, and it might be one of the essential targetable molecules in HCV patients who undergo DAA treatment and still suffer from a sustained HCV infection.

## PATIENTS AND METHODS

### Patients

This study enrolled 18 HCV GT-1b-infected hepatitis patients who had undergone OBV/PTV/r (OBV 25mg/day, PTV 150mg/day, r 100mg/day) oral administration for 12 weeks at Kagawa University Hospital (Kita-gun, Kagawa, Japan) between 2015 and 2017.

### The miRNA microarray for HCV GT-1b-infected serum

Each patient's serum sample was processed for total RNA extraction with the miRNeasy Mini Kit (Qiagen, Venlo, Netherlands) according to the manufacturer's instructions. The RNA samples typically showed *A*_260/280_ ratios of between 1.9 and 2.1, as shown by an Agilent 2100 Bioanalyzer (Agilent Technologies, Santa Clara, CA).

After RNA measurement with an RNA 6000 Nano kit (Agilent Technologies), the samples were labeled using a miRCURY Hy3/Hy5 Power labeling kit (Exiqon, Vedbaek, Denmark) and were hybridized on a human miRNA Oligo chip10, ver. 14.0 (Toray Industries, Tokyo). Scanning was conducted with a 3D-Gene Scanner 3000 (Toray). We used 3D-Gene extraction ver. 1.2 software (Toray) to read the raw intensity of the image. To determine the change in miRNA expression, we analyzed the raw data via GeneSpringGX ver. 10.0 (Agilent Technologies). All samples were frozen at –80 °C within 4 hr of collection and thawed just before analysis.

### Real time RT-PCR for quantifying circulating miRNA

Circulating miRNA was extracted from 200 μl of serum sample using the Qiagen miRNeasy serum-plasma kit (Qiagen, Tokyo) according to the manufacturer's instructions. RNA was reverse transcribed using TaqMan MicroRNA Reverse Transcription kit (Life Technologies Japan, Tokyo). *Caenorhabditis elegans* miR-39 (cel-miR-39) was spiked in each sample as a control for the extraction and amplification steps. Serum miRNA was amplified using primers and probes provided by Applied Biosystems (Foster City, CA, USA) by the TaqMan MicroRNA assay, according to the manufacturer's instructions. We calculated the relative expression of serum miRNA using the comparative cycle threshold (CT) method (2^-ΔΔCT^) with spiked cel-miR-39 as a normalized internal control.

### Cell culture

The human hepatocellular carcinoma cell line Huh7 was obtained from the Japanese Collection of Research Bioresources (JCRB) Cell Bank and transported to our laboratory. The Huh7 cell line was authenticated by the JCRB Cell Bank using short tandem repeat polymerase chain reaction (PCR). All cell lines were grown in RPMI-1640 medium (Gibco Invitrogen, Carlsbad, CA, USA) supplemented with 10% fetal bovine serum (FBS) and penicillin-streptomycin (100 mg/L; Invitrogen) at 37°C in a humidified atmosphere containing 5% CO_2_.

### Gene transfection

HCV genomic replicon was stably transfected into Huh-7 cells (NNC#2 cells). The gene transfection miR-636 mimic, the miR-636 inhibitor, and negative control miRNA were obtained from Thermo Scientific (Waltham, MA, USA). Huh-7 NNC#2 cells were seeded in six-well plates. After 24 hr, the Huh-7 NNC#2 cells were transfected with the miR-636 mimic, miR-636 inhibitor, or negative control miRNA at a final concentration of 10 nM using Lipofectamine RNAiMAX (Invitrogen, Grand Island, NY, USA). After 24 hour and 48 hour incubations, the cells were harvested and washed with ice-cold phosphate-buffered saline (PBS) for subsequent analysis.

### Statistical analysis

Replicate data were consolidated into two groups: those from serum samples obtained before OBV/PTV/r treatment, and those from serum samples obtained after OBV/PTV/r treatment. Those data were organized by using the hierarchical clustering functions in the GeneSpring software. Hierarchical clustering was done by the use of the clustering function (condition tree) and Pearson's correlation as a distance metric. We conducted a U-test and regarded p-value <0.0001 as a significant difference for the array analysis to search for the miRNAs that varied most prominently across the groups. Only changes >50% for at least one of the time points for each sample were considered significant. All of the analyzed data were scaled by global normalization. The statistical significance of differentially expressed miRNAs was analyzed by Student's t-test. All analyses were conducted using computer-assisted JMP8.0 (SAS Institute, Cary, NC, USA). A paired analysis between the groups was conducted using Student's t-test. A p-value <0.05 was considered to indicate a significant difference between groups.

## Abbreviations

ALT: alanine aminotransferase
DAA: direct-acting antiviral agent
GT-1b: genotype 1b
HCV: hepatitis C virus
OBV: ombitasvir
PTV: paritaprevir
R: ritonavir
miR-636: microRNA-636
cel-miR-39: cel-microRNA-39,
RQ: relative quantification
